# Shuang-Huang-Lian injection induces an immediate hypersensitivity reaction via C5a but not IgE

**DOI:** 10.1038/s41598-018-21843-7

**Published:** 2018-02-23

**Authors:** Yuan Gao, Rui Hou, Yixin Han, Qiaoling Fei, Runlan Cai, Yun Qi

**Affiliations:** Institute of Medicinal Plant Development, Chinese Academy of Medical Sciences & Peking Union Medical College, Beijing, 100193 China

## Abstract

Among traditional Chinese medicine injections, intravenous Shuang-Huang-Lian (IV-SHL) has the highest incidence of injection-induced immediate hypersensitivity reactions (IHRs). The precise mechanisms of IV-SHL-induced IHRs remain ambiguous. In this study, we investigated the mechanisms of SHL injection (SHLI)-induced IHRs. Our data showed that serum total IgE and mouse mast cell protease 1 (MMCP1) levels were higher in the SHLI antiserum; however, these effects of SHLI disappeared in the antibiotic-treated mice. SHLI caused intraplantar vasopermeability and shock during the first local or systemic injection. SHLI-induced nonallergic IHRs were attributed to its intermediate fraction F2 (the extract of *Lonicerae Japonicae Flos* and *Fructus forsythiae*), and could be blocked by antagonists for histamine or C5a, rather than PAF or C3a. Eight constituents of F2 were able to directly activate C5 to promote local vasopermeability at the mg/mL level. In conclusion, SHLI-induced IHRs are not mediated by IgE. SHLI or its F2 can directly activate blood C5. Subsequently, C5a is likely to provoke histamine release from its effector cells (e.g., mast cells and basophils), indicating that histamine is a principal effector of IHRs induced by SHLI.

## Introduction

Shuang-Huang-Lian (SHL), a modern antimicrobial formulation comprising alcohol-water extracts of three herbs (*Lonicerae Japonicae Flos*, *Scutellariae Radix*, and *Fructus Forsythiae*), is officially recorded in the Chinese Pharmacopoeia and is approved for production by the Chinese Food and Drug Administration (CFDA)^1^. Since 1973, intravenous SHL (IV-SHL) has been widely used in China to treat respiratory infection when an oral preparation is ineffective^2^. However, despite its benefits^3^, IV-SHL has been recorded to induce severe immediate hypersensitivity reactions (IHRs), namely anaphylaxis^4^, at a very low frequency^5^. Therefore, the CFDA has raised an alarm regarding this potentially life-threatening outcome^6^.

In China, about four hundred million patients are treated with traditional Chinese medicine injections (TCMI) per year. Adverse drug reactions (ADRs) have been reported with an incidence of approximately 1.51%^7,8^, representing around 67.3% of ADRs occurred within 1 h which belonged to IHRs^9^. Of these, IV-SHL has the highest incidence of ADRs^10,11^. Of the 11001 patients administered with IV-SHL, 182 (1.65%) had cutaneous and mucosal allergies (e.g., urticaria and rashes), while 14 (0.127%) suffered anaphylaxis^12^. Consequently, many researchers have focused on evaluating IHRs caused by IV-SHL. Some reports suggested that SHL injection (SHLI) significantly increased rat serum total IgE (tIgE) level^13^; whereas, others reported that SHLI could cause nonallergic IHRs (NA-IHRs)^14^. However, to design new strategies to prevent or treat SHLI-induced IHRs, the precise mechanism should be determined. To achieve this, we carried out a systemic study of the mechanisms of SHLI-induced IHRs. The two main possible mechanisms are IgE-mediated responses and complement activation-related pseudo-allergy (CARPA)^15^. Our results showed that the mechanism of SHLI-induced IHRs is CARPA rather than an IgE-mediated response. Our data indicated that SHLI can directly activate blood C5, which might subsequently stimulate its effector cells (e.g., mast cells and basophils) to release histamine^16^, which is the principal effector of IHRs induced by SHLI.

## Methods

### Materials and reagents

SHLI and its two intermediate fractions (F1, the extract of *Scutellariae Radix*; F2, the extract of *Lonicerae Japonicae Flos* and *Fructus Forsythiae*) were prepared by Duoduo Pharmaceutical Co., Ltd. (Jiamusi, Heilongjiang, China) according to the Chinese Pharmacopoeia^1^. Compound 48/80 (C48/80), propranolol, CV3988, triprolidine, globulins, cimetidine, and SB290157 were purchased from Sigma-Aldrich (St Louis, MO, USA). PMX53 was from GL Biochem Ltd. (Shanghai, China). Mouse tIgE ELISA kit was from Biolegend Co. (San Diego, CA, USA). Rehydragel^®^ aluminum adjuvant was from General Chemical (Parsippany, NJ, USA). Anti-human CD54-allophycocyanin (APC) antibody and its REA control-APC were from Miltenyi Biotec. (Bergisch Gladbach, German). Human C5a ELISA kit, fluorescein isothiocyanate (FITC)-conjugated mouse anti-human CD86 antibody and its isotype control were from BD Biosciences (San Diego, CA, USA). Gentamicin, ampicillin, 2-methyl-5-nitroimidazole-1-ethanol, and fradiomycin were from TCI Chemicals (Tokyo, Japan). Vancomycin was obtained from BBI Life Sciences Corporation (Shanghai, China). The mouse mast cell protease 1 (MMCP1) ELISA kit was from Invitrogen (San Diego, CA, USA). Protein G PLUS-Agarose was from Santa Cruz Biotechnology, Inc. (SantaCruz, CA, USA).

### Cells and animals

The human mast cell line LAD2 (from Michael D. Gershon, MD, Columbia University, USA) was a gift from Prof. Renshan Sun (the Third Military Medical University, Chongqing, China). The human THP-1 cell line was purchased from the Institute of Basic Medical Sciences, Chinese Academy of Medical Sciences (Beijing, China). Balb/c mice (female, 18–20 g) and SD rats (female, 160–180 g) were from Vital River Experimental Animal Services (Beijing, China). All the experiments were carried out according to the National Institutes of Health Guide for Care and Use of Laboratory Animals and approved by the Animals Ethics Committee of the IMPLAD of Chinese Academy of Medical Sciences.

### Preparation of shrimp tropomyosin (ST)

ST from *Metapenaeus ensis* was extracted and purified using an isoelectric precipitation method as previously described^17^.

### Preparation of ST or SHLI antisera

Balb/c mice were injected (i.p.) weekly with aluminum adjuvant (100 μL/mouse) containing SHLI (200 μL/mouse) or ST (60 μg/mouse). ST was used as a positive control allergen. The negative control (NC) mice were treated with aluminum adjuvant containing an equal volume of normal saline (NS). Five weeks later, the mice were sacrificed and the antisera were collected for subsequent assays.

### Measurement of tIgE and ST specific IgE (sIgE) in the antisera

Serum tIgE level was assayed using a commercial mouse tIgE ELISA kit. The levels of sIgE were measured as previously described, with some modifications^18^. Briefly, IgG in the serum were removed by Protein G PLUS-Agarose, according to the manufacturer’s instructions. The 96-well microtiter plates were coated with test constituent-bovine serum albumin (BSA) conjugate or ST (10 μg/mL, 100 μL/well) in coating buffer (0.05 M carbonate buffer, pH 9.6). After overnight incubation at 4 °C, plates were washed 4 times with phosphate-buffered saline (PBS)/0.05% Tween 20 and blocked with 1% BSA-PBS at 37 °C for 1 h. After washing, the IgG-free serum samples were added to the plates and incubated overnight at 4 °C. 100 μL of the HRP-labeled rat anti-mouse IgE antibody was added 4 times washing later. The plates were incubated at 37 °C for 1 h. The reactions were developed with 3,3′,5,5′-tetramethylbenzidine for 5 min at 37 °C and stopped using 100 μL of 2 M H_2_SO_4_. The optical density (OD) was read at 450 nm.

### Preparation of antibiotic-treated mice

The mice were fed autoclaved water with or without ampicillin (0.5 mg/mL), gentamicin (0.5 mg/mL), metronidazole (0.5 mg/mL), neomycin (0.5 mg/mL), and vancomycin (0.25 mg/mL) continuously *via* a water bottle. In the event of poor animal hydration, we supplemented control and antibiotic water with artificial sweetener^19^. Five weeks later, the mice were sacrificed, and the serum tIgE and MMCP1 levels were measured using ELISA.

### Human cell line activation test (h-CLAT) on THP-1 cells

According to previously described method^20^, THP-1 cells were treated with 34 constituents (50 μg/mL) for 24 h at 37 °C and their effects on CD54 and CD86 expression on the cell surface were determined by flow cytometry. Isotype control and unstained cells were used as the negative control (NC)^21^.

### Preparation of hapten-BSA conjugate

Forsythiaside A-BSA, arctigenin-BSA, and baicalein-BSA conjugates were prepared *via* a Mannich-type reaction, based on their active hydrogen, according to a previously described method^22^. Oleanolic acid is able to directly couple with BSA based on its free carboxyl.

### Passively sensitized animals

Passive cutaneous anaphylaxis (PCA) in rats was performed according to a previously described method with some modifications^23^. The rats were anesthetized and received an intradermal injection of 100 μL of SHLI antiserum (or 1/200 dilution of ST antiserum). Twenty-four hours later, the rats were injected via their tail vein with 1 mL of 5 mg/mL Evans Blue containing 1 mg forsythiaside A-BSA, arctigenin-BSA, oleanolic acid-BSA, baicalein-BSA conjugate, or 500 μg ST, respectively. The resultant blue spots in the dorsal inboard skin were scored 1 h later.

In the MMCP1 assay, Balb/c mice were primed (i.v.) with NC serum (500 μL/mouse), ST antiserum (10 μL/mouse), or SHLI antiserum (500 μL/mouse). Twenty-four hours later, the mice were challenged (i.v.) with ST (200 μg/mouse) or SHLI (200 μL/mouse). The NC mice were treated with NS. Four hours later, whole blood was obtained and the MMCP1 concentration in the serum was measured using ELISA^24^.

### Evans Blue extravasation assays

Evans Blue extravasation in mice hind paws was measured as previously described^25^. For the rat cutaneous Evans Blue extravasation assay, animals were subcutaneously injected with 100 μL of test substances and then immediately intravenously injected with 5 mg/mL of Evans Blue (1 mL). Fifteen minutes later, the rats were sacrificed and the resultant blue spots in the dorsal inboard skin were scored.

### Anaphylactoid shock assay

Anaphylactoid shock was assessed by rectal thermometry^26^. To detect the effects of test substances on the temperature of normal mice, Balb/c mice were intravenously injected with the test substances, including NS, C48/80 (40 μg/mouse), SHLI (0.5 mL/mouse), F1 (4.81 mg/mouse), or F2 (16.5 mg/mouse), respectively. Thirty minutes later, the rectal temperature was measured. To increase the severity of anaphylaxis^27^, the mice were pretreated (i.v.) with propranolol (35 μg/mouse). Twenty minutes later, the mice were challenged (i.p.) with the test substances. After 30 min, the rectal temperature was measured. For the platelet activating factor (PAF) or histamine antagonist experiments, 1 mg/mL of antagonist (CV3988, triprolidine, and cimetidine, 200 μL/mouse) was intraperitoneally injected into the mice 10 min before the propranolol pretreatment.

### Complement activation assay *in vitro*

Human whole blood and plasma from healthy volunteers were obtained with written informed consent. The plasma was prepared by centrifuging blood that had been collected in EDTA-coated tubes and was immediately used or frozen at −80 °C until use. Plasma (100 μL, 1:100 dilution) was mixed with 4 μL of CaCl_2_ (1 M), 4 μL of MgCl_2_ (1 M), and 10 μL of test substances, and then incubated at 37 °C. After 20 min, the reaction was stopped using 22 μL of EDTA (0.5 M, pH 8.0) and cooling to 0 °C. Tween-80 was used as a positive control^28^. The level of C5a in the plasma was determined using a commercial ELISA kit according to the manufacturer’s instruction.

### Statistical analysis

Data were expressed as the means ± SD from at least three independent experiments and were analyzed by a one-way analysis of variance (ANOVA). A Student *t*-test was used when only two groups were compared. A difference with a *P*-value < 0.05 was considered statistically significant.

## Results

### SHLI-induced IHRs are not mediated by IgE

#### Serum tIgE and MMCP1 levels are higher in the SHLI antiserum

**Figure 1:**
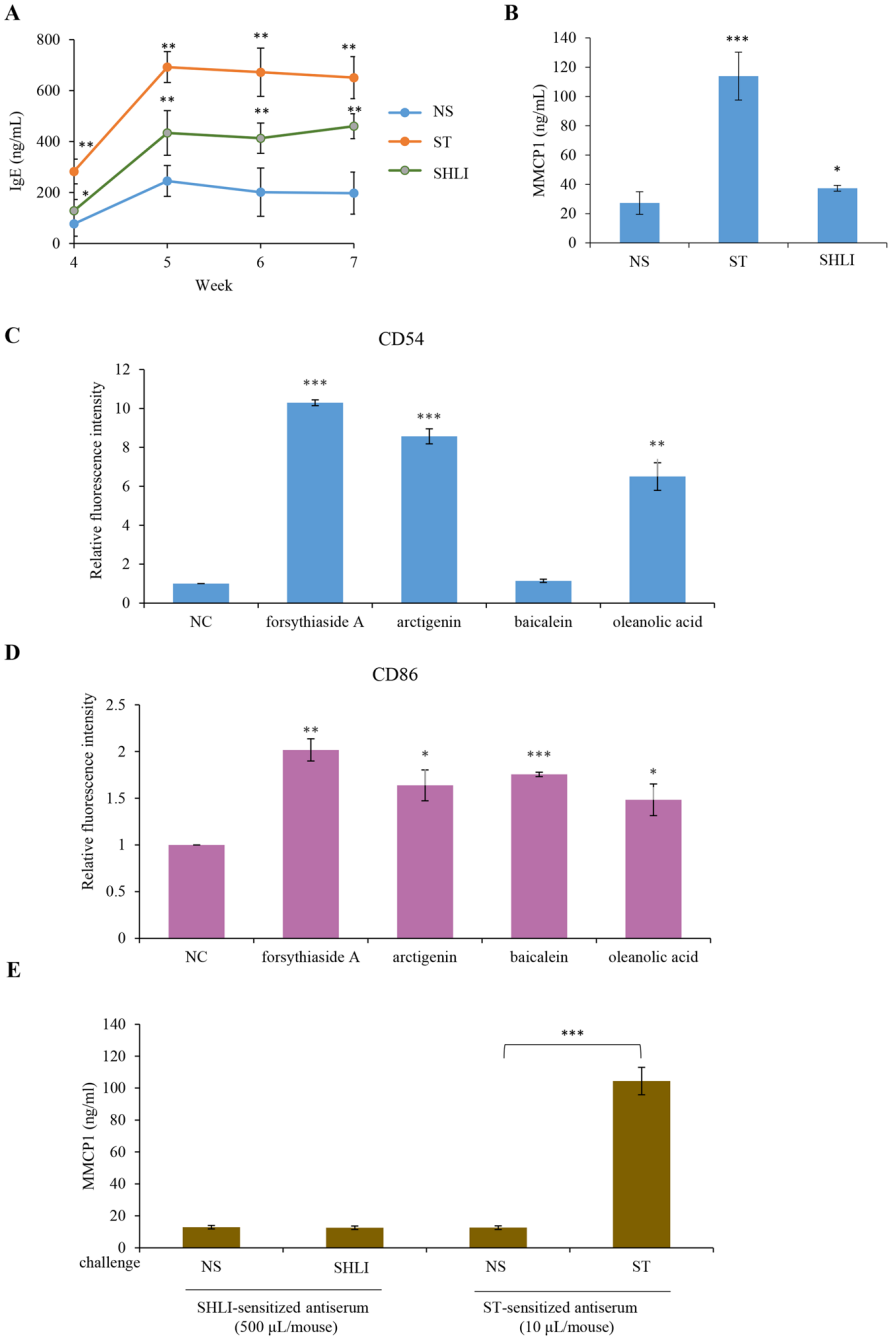
(**A**) SHLI elevates mouse serum tIgE levels 4–7 weeks after immunization (n = 8). The mice were injected weekly (i.p.) with aluminum adjuvant (100 μL/mouse) containing SHLI (200 μL/mouse) or ST (60 μg/mouse). The serum tIgE level was determined using a commercial ELISA kit. (**B**) SHLI causes an increase in mouse serum MMCP1 5 weeks after immunization (n = 8). The mice were injected weekly (i.p.) with aluminum adjuvant (100 μL/mouse) containing SHLI (200 μL/mouse) or ST (60 μg/mouse). The serum MMCP1 level was determined using a commercial ELISA kit. ^*^P < 0.05, ^**^P < 0.01 and ^***^P < 0.001 *vs*. NS. (**C,D**) Four constituents in SHLI increase THP-1 cells CD54 (**C**) and/or CD86 (**D**) levels. THP-1 cells were treated with 4 constituents (50 μg/mL) for 24 h at 37 °C and then stained with anti-human CD54-APC or anti-human CD86-FITC antibodies for 15 min at 25 °C. The expression of CD54 and CD86 on THP-1 cells was assayed by flow cytometry. Isotype control and unstained cells were used as NC. ^*^P < 0.05, ^**^P < 0.01 and ^***^P < 0.001 *vs*. the NC. (**E**) The serum MMCP1 concentration does not increased in the mice passively sensitized by SHLI antiserum. Balb/c mice were primed (i.v.) with NC serum (500 μL/mouse) or ST antiserum (10 μL/mouse) or SHLI antiserum (500 μL/mouse). Twenty-four hours later, the mice were challenged (i.v.) with ST (200 μg/mouse) or SHLI (200 μL/mouse). The NC mice were treated with an equal volume of NS. Four hours later, the whole blood was obtained and MMCP1 concentration in the serum was measured using an ELISA. ^***^P < 0.001.

IgE-mediated IHRs are the most common allergic IHRs^29^. To amplify the possible Th2 response induced by SHLI, an aluminum adjuvant was used during mouse immunization. As shown in Fig. 1A, serum tIgE levels were significantly elevated by ST and SHLI, 4 weeks after intraperitoneal immunization, and peaked and stabilized between 5–7 weeks. Consistently, serum MMCP1, a specific marker for IgE-mediated allergy^24^, was significantly increased in the SHLI antiserum from the mice sensitized by SHLI for 5 weeks (Fig. 1B). These results seemingly suggested that SHLI might induce IgE-mediated IHRs.

#### Four suspicious allergens are not the haptens for IgE

The tIgE is the summation of sIgEs against different antigens, and MMCP1 is a specific marker for type I hypersensitivity. The fact that SHLI elevated serum tIgE and MMCP1 levels prompted us to look for the potential allergenic components from SHLI. To efficiently and quickly screen the possible haptens, the h-CLAT method was used^20^. Thirty-four available constituents (50 μg/mL; Table S1) in SHLI were identified based on CD54 or/and CD86 expression in THP-1 cells, of which 4 constituents (forsythiaside A, arctigenin, oleanolic acid, and baicalein) might possess sensitization potential (Fig. 1C,D). We then synthetized their respective holoantigens (hapten-BSA conjugates) to measure their respective sIgE in the SHLI antiserum using ELISA (*in vitro*). Unexpectedly, no sIgE was detected, while anti-ST sIgE was markedly high in the parallel experiments (data not shown). Consistently, these conjugates also could not result in blue spots in a PCA assay (data not shown), suggesting that 4 suspicious allergens are not the haptens for IgE.

There are two possibilities for the above phenomenon: 1) The haptens for IgE are not included in the 4 constituents or in the other 30 constituents; 2) SHLI does not cause IgE-mediated IHRs. To clarify this issue, serum MMCP1 of passively sensitized mice was determined. Unlike ST, SHLI could not increase the serum MMCP1 level (Fig. 1E), suggesting that SHLI-induced IHRs are not mediated by IgE.

### SHLI fails to elevate serum tIgE and MMCP1 in antibiotic-treated mice

**Figure 2:**
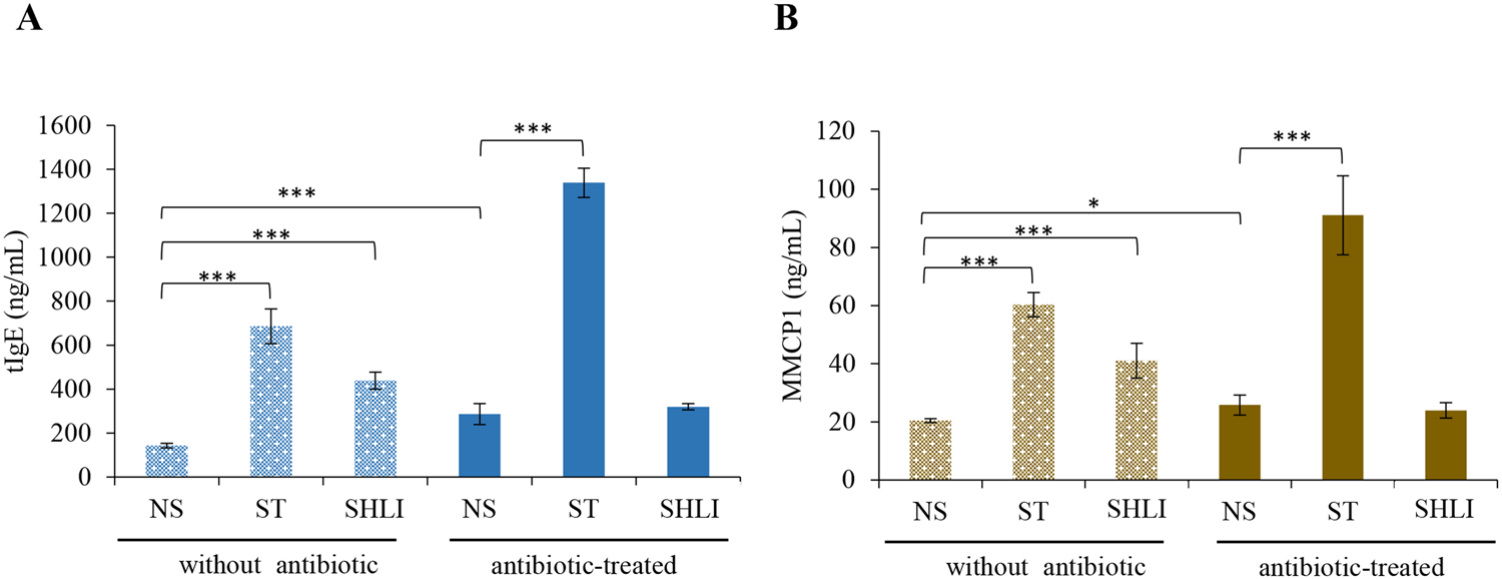
SHLI fails to elevate serum tIgE (**A**) and MMCP1 (**B**) levels in antibiotic-treated mice (n = 8). We fed mice autoclaved water with or without ampicillin (0.5 mg/mL), gentamicin (0.5 mg/mL), metronidazole (0.5 mg/mL), neomycin (0.5 mg/mL), and vancomycin (0.25 mg/mL) continuously *via* a water bottle. In the event of poor animal hydration, we supplemented control and antibiotic water with artificial sweetener. Five weeks later, the mice were sacrificed, and the serum tIgE and MMCP1 levels were measured by ELISA. ^*^P < 0.05 and ^***^P < 0.001.

Given that commensal bacteria-derived signals can limit serum IgE levels^19,30^, together with the fact that SHL is an antimicrobial^31^, we speculated that SHLI-induced serum tIgE and MMCP1 elevation would be relevant to its suppressive effect on commensal bacteria-derived signals. To confirm this hypothesis, antibiotic-treated mice, in which the antimicrobial activity of SHLI might be completely restricted, were used^19^. As expected, the SHLI-mediated elevation of serum tIgE and MMCP1 (Fig. 1A,B) disappeared, while that of ST was not attenuated (Fig. 2).

### SHLI contributes to NA-IHRs by activating C5

IHRs can be allergic- as well as nonallergic-mediated^32^; therefore, we next evaluated whether SHLI could induce NA-IHRs. Our study had demonstrated that SHLI could not directly induce human mast cells LAD2 degranulation (data not shown). In fact, our previous experiment showed that SHLI could markedly dampened C48/80-induced degranulation in mast cells^17^. Therefore, in the present study, we evaluated whether SHLI could induce local and systemic NA-IHRs *in vivo*.

#### SHLI induces vasopermeability during the first intraplantar injection

**Figure 3:**
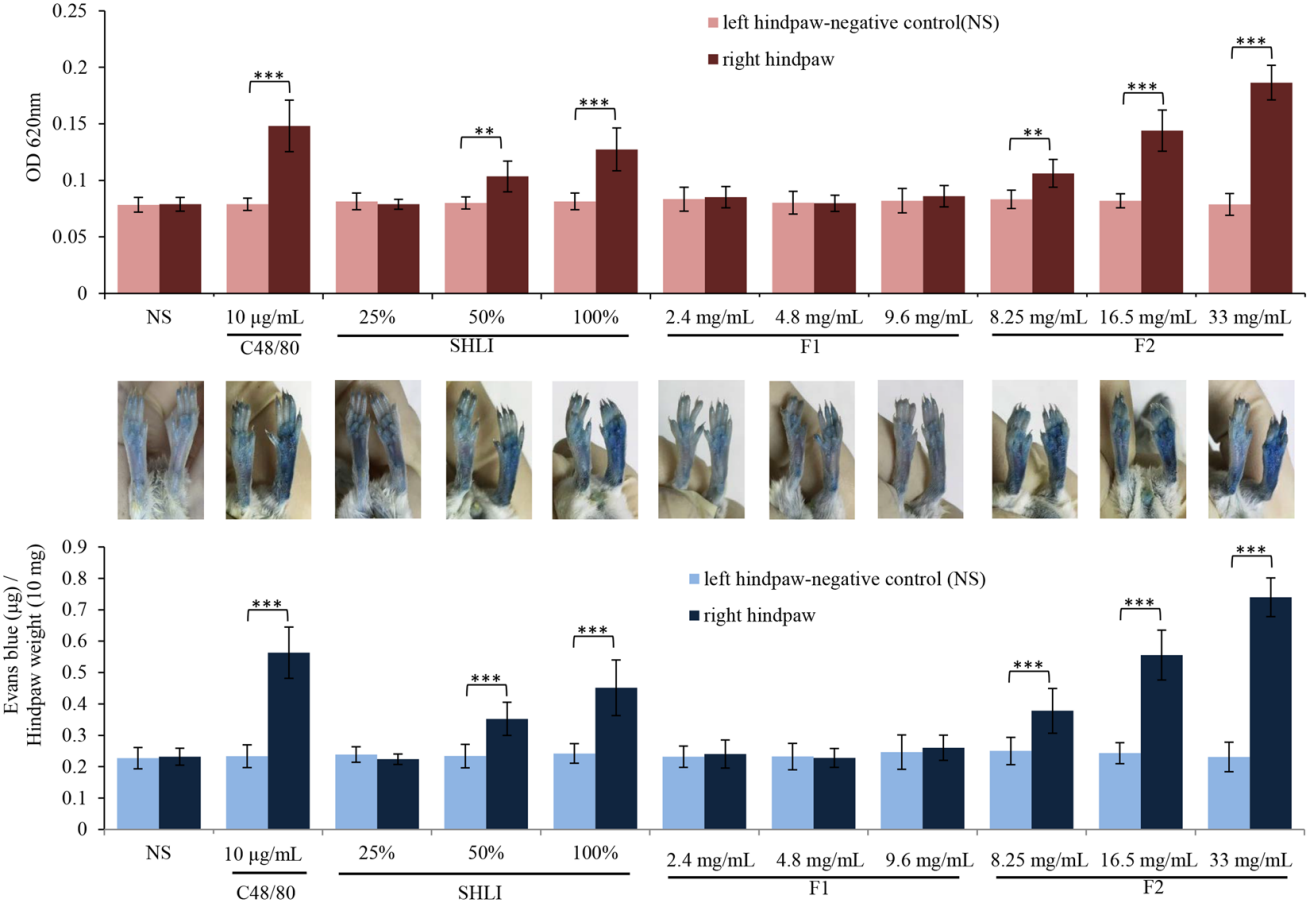
SHLI induces vasopermeability (Evans Blue extravasation) during the first intraplantar injection (n = 6). Fifteen minutes after induction of anesthesia (50 mg/kg of pentobarbital), mice were injected intravenously (i.v.) with 50 μL of 12.5 mg/mL Evans Blue. Five minutes later, 5 μL of the test substance (C48/80, SHLI, F1, or F2) was administered by intraplantar injection in the right paw and NS was administered in the left paw. Thirty minutes later, the mice were sacrificed and the paw tissue was collected, dried for 24 h at 50 °C, and weighed. Evans Blue was extracted by a 24 h incubation in formamide at 50 °C, and the OD value was read at 620 nm. ^**^P < 0.01 and ^***^P < 0.001.

We first determined the local allergenicity of SHLI using a sensitive hindpaw Evans Blue extravasation assay. As shown in Fig. 3, the positive control C48/80 (10 μg/mL), SHLI (50% and 100%), and its intermediate fraction F2 (8.25 mg/mL–33 mg/mL; 33 mg/mL was the equivalent concentration in 100% SHLI) significantly induced vasopermeability during the first intraplantar injection, while F1 (9.6 mg/mL was the equivalent concentration in 100% SHLI) had no effect, indicating that F2 might be the major contributor in SHLI that induces local NA-IHRs.

#### F2 of SHLI induces anaphylactoid shock by histamine, rather than PAF

**Figure 4:**
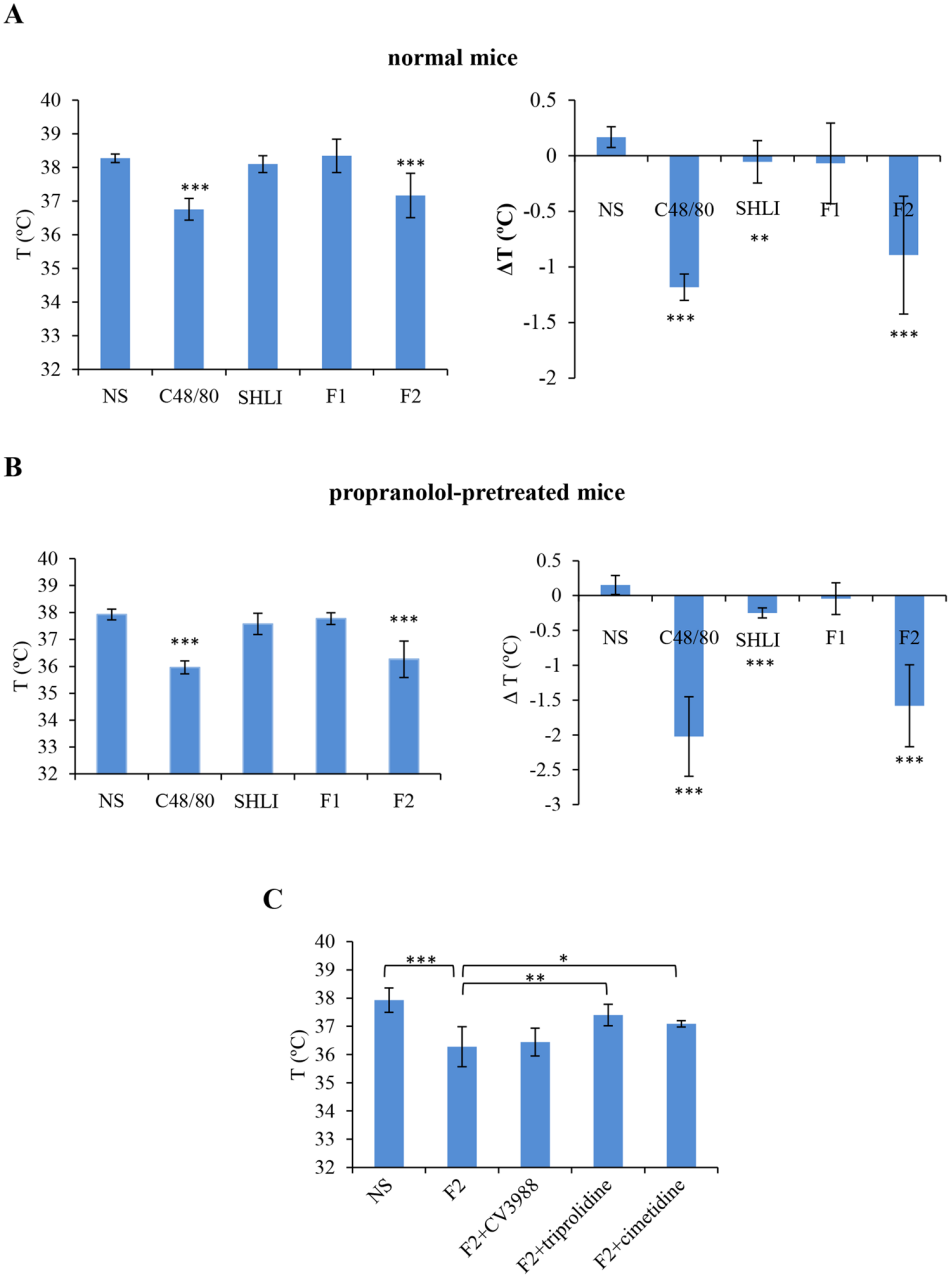
(**A**) F2 of SHLI induces anaphylactoid shock in normal mice (n = 6). The mice were intravenously injected with NS, C48/80 (40 μg/mouse), SHLI (0.5 mL/mouse), F1 (4.81 mg/mouse), or F2 (16.5 mg/mouse). Thirty minutes later, the rectal temperature was measured. ^**^P < 0.01 and ^***^P < 0.001 *vs*. NS. (**B**) F2 of SHLI induces anaphylactoid shock in propranolol-pretreated mice (n = 6). The mice were pretreated intravenously (i.v.) with propranolol (35 μg/mouse). Twenty minutes later, the mice were intraperitoneally injected with NS, C48/80 (40 μg/mouse), SHLI (0.5 mL/mouse), F1 (4.81 mg/mouse), or F2 (16.5 mg/mouse). Thirty minutes later, the rectal temperature was measured. ^***^P < 0.001 *vs*. NS. (**C**) F2 of SHLI-induced shock is histamine dependent (n = 6). The mice were intraperitoneally treated with 0.2 mg/mouse of CV3988 (a PAF antagonist), triprolidine (an H1 antagonist), and cimetidine (an H2 antagonist), and then (10 min later) injected (i.v.) with propranolol (35 μg/mouse). Twenty minutes later, the mice were challenged (i.p.) with F2 (16.5 mg/mouse). Thirty minutes later, the rectal temperature was measured. ^*^P < 0.05, ^**^P < 0.01 and ^***^P < 0.001.

We next evaluated whether SHLI could cause anaphylactoid shock (detected as hypothermia^26^). Mice were made more sensitive to SHLI-induced shock by pretreatment with propranolol, which does not induce shock by itself, but can increase the severity of shock^27,33^. As shown in Fig. 4A,B, C48/80 and F2 contributed to obvious hypothermia in both normal and propranolol-pretreated mice, while F1 has no effect on the mice’s temperature. SHLI significantly induced hypothermia only by comparing the temperature difference (ΔT °C) before and after treatment. Given the fact that F2 showed a more obvious effect, we chose F2 instead of SHLI in the subsequent antagonist study.

Mast cells, basophils, and macrophages contribute predominantly to the pathogenesis of anaphylaxis through their secretion of histamine or/and PAF^26,34^. An experiment performed using specific antagonists for PAF (CV3988), histamine H1 receptor (triprolidine) and H2 receptor (cimetidine) demonstrated that F2-induced shock was substantially ameliorated by triprolidine and cimetidine, but not CV3988 (Fig. 4C), indicating that histamine, rather than PAF, was the principal effector in F2-induced systemic anaphylaxis.

#### SHLI promotes local and systemic NA-IHRs by anaphylatoxin C5a

**Figure 5:**
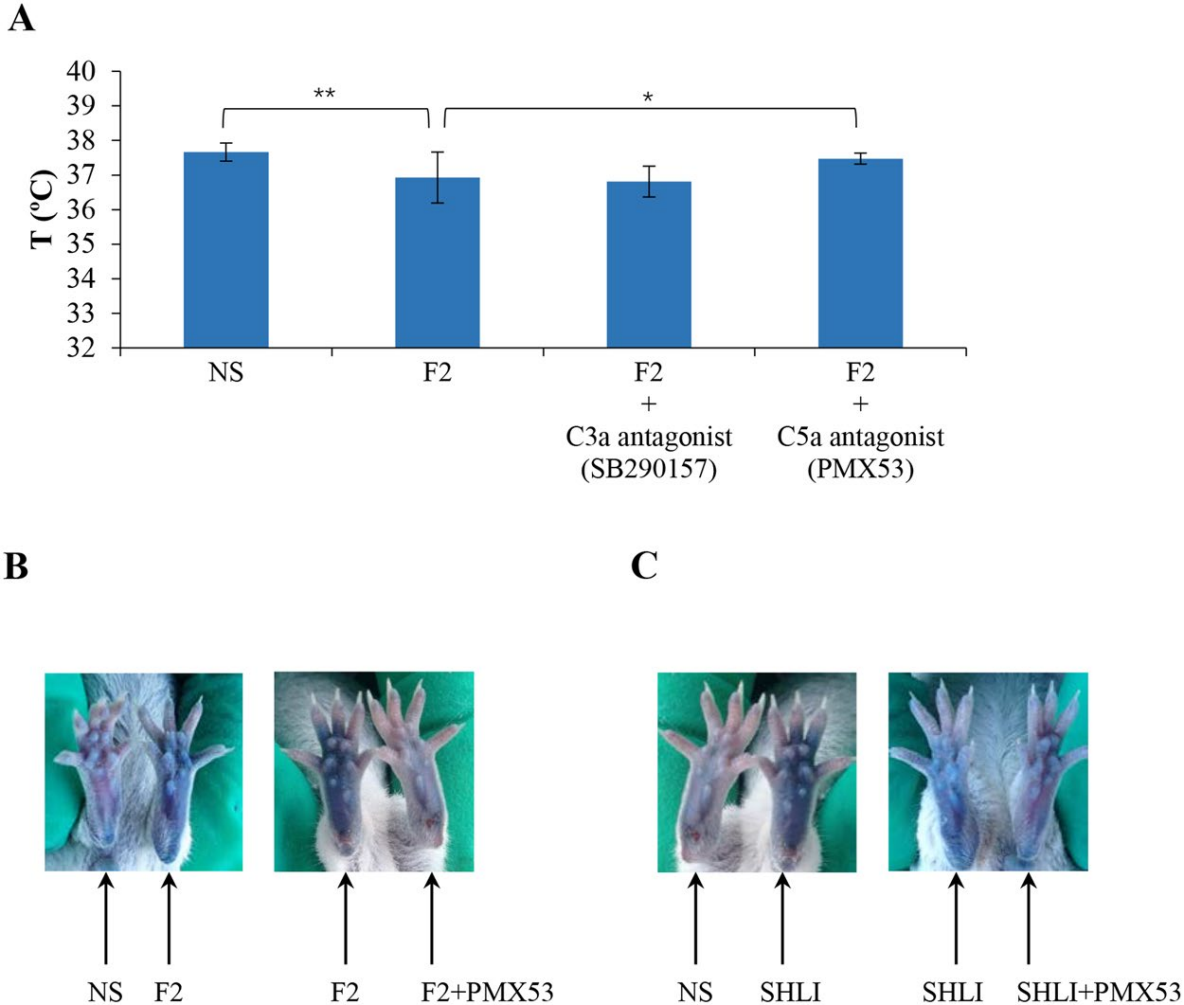
SHLI-induced NA-IHRs is mediated by C5a. (**A**) C5a antagonist PMX53 can block F2-induced shock (n = 10). The mice were pretreated with propranolol (i.v., 35 μg/mouse) 10 min after intraperitoneally injected with SB290157 (a C3a antagonist, 30 mg/kg) or PMX53 (a C5a antagonist, 1 mg/kg). Twenty minutes later, the mice were challenged (i.p.) with F2 (16.5 mg/mouse). Thirty minutes later, the rectal temperature was measured. ^*^P < 0.05 and ^**^P < 0.01. (**B**) Representative images of Evans Blue extravasation of mouse paw induced by F2 and blocked by PMX53 (n = 6). Fifteen minutes after induction of anesthesia (50 mg/kg of pentobarbital), mice were intraplantarly injected with 10 μL of PMX53 (1 mg/mL). Twenty minutes later, the mice were injected (i.v.) with 50 μL of 12.5 mg/mL Evans Blue. Five minutes later, 5 μL of F2 (33 mg/mL), or NS was administered by intraplantar injection in the paw. Thirty minutes later, the mice were sacrificed and the paws were photographed. (**C**) Representative images of Evans Blue extravasation of mouse paw induced by SHLI (10 μL/paw) and blocked by PMX53 (n = 6).

The above observations indicated that SHLI or F2 are likely to promote local and systemic NA-IHRs by inducing the release of histamine, a mast cell-derived mediator. Anaphylatoxin C3a or C5a can activate mast cells and basophils to secrete histamine^35^. Thus, it is possible that SHLI induces NA-IHRs via the activation of complement. Consequently, we used a C3a antagonist (SB290157) or a C5a antagonist (PMX53) to antagonize F2-induced shock. The result showed that PMX53, but not SB290157, significantly attenuated F2-induced hypothermia (Fig. 5A). Next, in a hindpaw Evans Blue extravasation assay, F2- or SHLI-induced vasopermeability could be blocked by PMX53 (Fig. 5B,C), indicating SHLI’s induction of NA-IHRs was attributed to C5a, rather than C3a.

#### Eight constituents of F2 can induce local vasopermeability by directly activating C5

**Figure 6:**
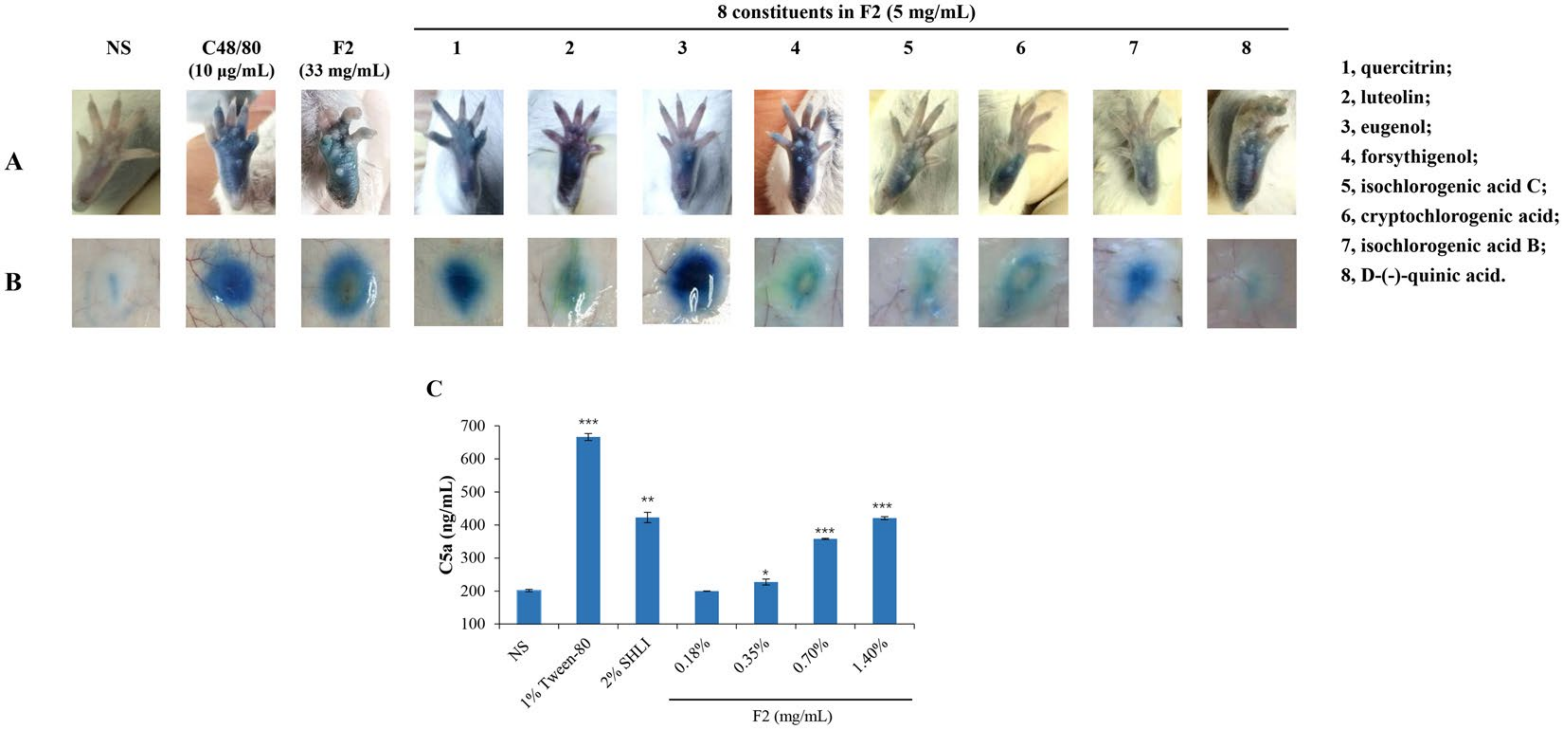
(**A**) Representative images of Evans Blue extravasation of mouse hindpaw induced by 8 allergenic constituents in F2. Fifteen minutes after induction of anesthesia (50 mg/kg of pentobarbital), mice were injected (i.v.) with 100 μL of 6.25 mg/mL Evans Blue in saline. Five minutes later, 15 μL of the test substance was administered by intraplantar injection in one paw. Fifteen minutes later, the mice were sacrificed and the paws were observed. (**B**) Representative images of Evans Blue extravasation of rat dorsal skin induced by seven allergenic constituents in F2. Rats were subcutaneously injected with 100 μL of test substances and then immediately injected (i.v.) with 5 mg/mL of Evans Blue (1 mL). Fifteen minutes later, the rats were sacrificed and the resultant blue spots were scored. (**C**) SHLI and F2 can activate C5 in plasma *in vitro*. The plasma was treated with test substances at the indicated concentrations and C5a level was determined by a commercial ELISA kit. ^*^P < 0.05, ^**^P < 0.01 and ^***^P < 0.001.

To identify the possible pseudoallergens in SHLI, we tested the effects of 29 available constituents (5 mg/mL) from F2 (Table S1) using Evans Blue extravasation assays in both mouse and rat models. Eight constituents (cryptochlorogenic acid, forsythigenol, isochlorogenic acid B and C, eugenol, quercitrin, D-(−)-quinic acid, and luteolin) significantly induced vasopermeability during the first intraplantar injection in mice (Fig. 6A); however, the effect of D-(−)-quinic acid in rats was not observed (Fig. 6B). Moreover, the effects of 8 constituents on intraplantar vasopermeability could also be blocked by the C5a antagonist PMX53 (photos not shown).

Next, we determined whether the 8 constituents could directly activate C5 *in vitro* using a commercial C5a ELISA kit. Except for luteolin, which was likely to interfere with the ELISA system, the other 7 constituents could directly activate C5 *in vitro* in a concentration-dependent manner; their half effective concentration (EC_50_) values were shown in Table S2. We also determined the effects of SHLI and F2. As expected, SHLI or F2 increased the C5a level in plasma in a concentration-dependent manner (Fig. 6C).

## Discussion

Although TCMI-induced ADRs occur at a very low frequency, anaphylaxis attracts great attention because of its life-threatening outcome. IV-SHL has the highest incidence of TCMI-induced IHRs. For this reason, many researchers have focused on evaluating IHRs induced by IV-SHL. The majority of them supported the view that IV-SHL could cause IgE-mediated IHRs^13,36–39^ although this was also questioned^40,41^, while only a few researchers reported that IV-SHL has the potential to cause an anaphylactoid reaction resulting from direct degranulation in mast cells or basophils^42,43^. Nevertheless, to date, the supporters of IgE-mediated IHRs have not identified the specific IgE for SHLI, and the supporters of anaphylactoid reaction cannot provide a convincing mechanism. Therefore, the mechanisms of IV-SHL-induced IHRs are a matter of debate. In the present study, we systemically researched the mechanisms of IHRs induced by IV-SHL.

IHRs, sub-divided into mild, moderate, or severe/life-threatening (namely anaphylaxis^4^), can be allergic- as well as nonallergic-mediated^32^. In mice, two immunological pathways have been defined: IgE-FcεRI mediates the classical pathway and IgG-FcγRIII mediates the alternative pathway^26,29,44^. Considerably more antibody and antigen are required to induce IgG- compared with IgE-mediated anaphylaxis^29^, which most likely reflects the much higher affinity of IgE binding to FcεRI than IgG binding to FcγRIII. By immunization with an aluminum adjuvant, SHLI significantly elevated the serum tIgE and MMCP1 levels (Fig. 1A,B), which is in agreement with a previous report^13^. The tIgE is the summation of sIgE against different antigens and SHLI prepared by water-extraction and alcohol-precipitation mainly comprises micromolecular compounds. Unexpectedly, screening by the h-CLAT method showed that 4 suspicious allergens were not the haptens for IgE. Moreover, after challenge by SHLI, the serum MMCP1 concentration of passively sensitized mice was not affected (Fig. 1E). These findings demonstrate that SHLI does not contribute to IgE-mediated IHRs. In fact, our data showed that the elevated effects of SHLI on serum tIgE and MMCP1 could be attributed to its antimicrobial activity (Fig. 2).

Clinically, IHRs induced by SHLI commonly occur after a first exposure^45^, conforming to the characteristics of NA-IHRs^25^. However, as a mitochondrial calcium uniporter agonist, SHLI can directly dampen mast cells or basophils degranulation^17^, suggesting that the IHRs induced by SHLI may occur indirectly. Thus, in this study, we evaluated SHLI-induced local and systemic NA-IHRs *in vivo*. Our data indicated that SHLI, predominantly its F2 fraction, contributed to local and systemic NA-IHRs (Figs 3 and 4A,B). Using specific antagonists^26,34^, we showed that histamine, rather than PAF, was the principal effector in SHLI-induced NA-IHRs (Fig. 4C). To our knowledge, PAF is the major mediator responsible for IgG/C3a-induced anaphylaxis in macrophages^26,27,46^, while histamine is a mast cell-derived mediator. Besides MrgprB2 or MrgprX2 on mast cells^25^, engagement of complement receptors with their ligands can also induce histamine release^47^. In fact, CARPA is a main class of drug-induced acute immune toxicity^15^. The complement-derived anaphylatoxins C3a and C5a act as cell activators by binding to their respective receptors, C3aR and C5aR. Anaphylatoxin receptors are widely expressed on the cells of myeloid origin, including mast cells, basophils and macrophages, etc. Unlike macrophages liberation PAF, histamine is released by mast cells and basophils^16^. Due to a very little amount of histamine released from basophils^34^, the major effector cells should be mast cells^48^. C5a has long been identified as a potent liberator of histamine^49^, our findings identified that SHLI promotes NA-IHRs by directly activating C5, thereby activating its effector cells to release histamine (Fig. 5). Notably, 8 constituents from F2 could directly activate C5 (Fig. 6 and Table S2) to promote local vasopermeability at the mg/mL level, which are far higher than their respective equivalent concentrations in SHLI, indicating that there might be a series of active ingredients similar to these 8 constituents that collectively activate C5 to induce NA-IHRs.

In summary, this is the first study to show that SHLI-induced IHRs is a CARPA, rather than an IgE-mediated allergy. SHLI-induces C5 activation and then provokes histamine release, which might be a principal effector of its IHRs. The results of the present study might lead to the design of new strategies to prevent or treat IHRs induced by IV-SHL.

## Electronic supplementary material

Supporting information
