# Supplementary material for: Shuang-Huang-Lian injection induces an immediate hypersensitivity reaction via C5a but not IgE
Source: Sci Rep. 2018 Feb 23;8:3572. doi: 10.1038/s41598-018-21843-7 (PMC5824823; doi:10.1038/s41598-018-21843-7)
Supplement: Supplementary file 1 — Supporting information [file 41598_2018_21843_MOESM1_ESM.pdf]

Shuang-Huang-Lian injection induces an immediate hypersensitivity reaction via C5a but not IgE

Yuan Gao, Rui Hou, Yixin Han, Qiaoling Fei, Runlan Cai and Yun Qi*

Institute of Medicinal Plant Development, Chinese Academy of Medical Sciences & Peking Union Medical College, Beijing, 100193, China

* Corresponding author.

Table S1 Herbal sources of the 34 available constituents in SHLI.

No.	Constituent	Source		
		Lonicerae Japonicae Flos	*Fructus Forsythiae*	*Scutellariae Radix*
1	neochlorogenic acid	+		
2	luteolin	+		
3	chrysophanol	+*		
4	oleanolic acid		+	
5	isopimpinellin	+*		
6	rutin	+	+	
7	cryptochlorogenic acid	+		
8	physcion	+*		
9	methyl salicylate		+	
10	D-(-)-quinic acid	+		
11	isochlorogenic acid A	+		
12	isochlorogenic acid B	+		
13	isochlorogenic acid C	+		
14	quercitrin	+		
15	quercetin	+	+	
16	chlorogenic acid	+		
17	eugenol	+		
18	forsythiaside B		+	
19	hyperin	+		
20	ursolic acid		+	
21	caffeic acid	+	+	
22	acetophenone		+	
23	forsythigenol		+	
24	forsythin		+	
25	forsythiaside E		+	
26	caffeoylquinic acid	+		
27	forsythiaside A		+	

28	arctiin	+	
29	arctigenin	+	
30	wogonin		+
31	baicalin		+
32	wogonoside		+
33	baicalein		+
34	scutellarin		+

* These constituents had been identified in the SHLI, but their herbal sources had not been confirmed.

Table S2 EC_{50} values of eight constituents in SHLI for complement C5 activation in plasma.

**Test substances**	$\mathbf{ED_{50}}$
Tween-80	0.085%
cryptochlorogenic acid	0.85 mg/mL
forsythigenol	0.65 mg/mL
isochlorogenic acid C	0.33 mg/mL
eugenol	0.52 mg/mL
quercitrin	1.16 mg/mL
isochlorogenic acid B	0.56 mg/mL
D-(-)-quinic acid	0.53 mg/mL
luteolin	ND*

*: Not detected. Luteolin can interfere with the assay system.
